# Facing reality: the growth of virtual reality and health sciences libraries^*^

**DOI:** 10.5195/jmla.2017.329

**Published:** 2017-10-01

**Authors:** Susan Lessick, Michelle Kraft

## INTRODUCTION

Virtual reality (VR) is an increasingly hot tech topic. Because VR may be the ultimate virtual project as defined by this column, replacing the real world with a simulated one, it is worthwhile to pause and reflect on its potential and practicality for health sciences libraries. Virtual reality is a computer technology that uses headsets to create an immersive, computer-generated simulation that allows end users to experience, move about in, and interact with this virtual world, shutting out the real world. Other tightly related technologies that are growing in popularity are augmented reality (AR), which overlays a three-dimensional (3D) environment with supplemental data such as graphics or sound, enabling end users to interact with both physical and virtual objects, and mixed reality (MR), which aims to combine both VR and AR, allowing the digital or VR world to interact in real world surroundings. These new “realities” can create unique experiences that expand learning opportunities and engagement for end users.

While the most prevalent uses of these technologies thus far have been in the consumer sector, tools for creating these applications are becoming easier to use and more affordable in the education and health care sectors. The *NMC Horizon Report,* which charts emerging technologies and uptake in higher education, notes that educational exposure to VR and AR is growing, and early research indicates that their use may have positive effects on student learning [1]. The report suggests that these visualization technologies are already transforming medical education and describes several innovative deployments in medical education settings, including the Augmentarium at the University of Maryland for surgery training and the development of a holographic medical anatomy curriculum at Case Western Reserve University, in partnership with the Cleveland Clinic. One only has to look at the “Virtual Reality News” section of the *Science Daily News* to see the growing interest and excitement surrounding VR in health care and education.

What do these developments mean for health sciences libraries? Health sciences libraries are well poised to play a bigger role in helping clinicians, faculty, students, and researchers discover and leverage the use of VR and AR tools. As VR and AR accelerate in health care and education, health sciences librarians need to keep apprised of these new formats as they will be responsible for managing and sharing this challenging new user-generated content. As more and more libraries serve as creation hubs for their users, librarians are well positioned to provide VR and AR resources, spaces, strategies, and connections to support the creative endeavors of their users—and to explore VR and AR applications for themselves and better understand their relevance in enhancing the user experience and improving and extending library services.

This year’s column profiles six different virtual projects that were selected by an advisory committee of technology experts after an annual call for projects in MLA-FOCUS and announcements to encourage submissions from all types of libraries. Each project provides a pathway for librarians to understand and effectively leverage new technologies to enhance library services.

The first project describes a librarian-lead VR service in an academic medical library that is representative of the shift that is taking place on campuses, as students are learning by *creating* rather than by solely consuming content. The startup VR service at the Claude Moore Health Sciences Library at the University of Virginia illustrates a new role for libraries in catalyzing user creativity by providing exposure to VR technology and technology-enabled spaces to support VR use by a broad range of users.

In Australia, librarians at Macquaire University creatively developed an engaging interactive game for undergraduate students to spark curiosity and instill evidence-based practice (EBP) knowledge and skills, while rewarding students for successfully completing learning tasks.

Another future-forward project, developed by librarians at the University of Houston, creatively uses data dashboards in a data literacy skills program that empowers students to interactively analyze health inequities in their own communities.

As scholarly records proliferate in different formats, the Galter Health Sciences Library & Learning Center at Northwestern University forged new strategies and a unique solution to this challenge by launching a next-generation repository that provides access and stewardship for nontraditional scholarly outputs.

The final two projects spotlight the technical skills and resourcefulness of librarians as they strive to find practical solutions to common problems. MEDLINE Transpose, a freely available, open source tool for researchers that automatically converts PubMed and Ovid interfaces, was developed by two librarians who hand-coded the tool themselves to simplify complex search processes. Lastly, librarians at the Southern Illinois University School of Medicine Library smartly developed an analytics database using locally licensed software to gain a better understanding of patron interactions, which they used for external reports and library decision making.

We hope these imaginative virtual projects will illuminate new ideas and inspire other librarians to consider, utilize, develop, and spread new technology solutions that reshape library services, making them more responsive, nimble, and effective. Please consider sharing your own virtual project in future columns. Submissions, suggestions, and questions should be directed to Susan Lessick, AHIP, FMLA.
