# Supplementary material for: Facing reality: the growth of virtual reality and health sciences libraries
Source: J Med Libr Assoc. 2017 Oct 1;105(4):407–17. doi: 10.5195/jmla.2017.329 (PMC5624433; doi:10.5195/jmla.2017.329)
Supplement: Related texts

# Other: Imagination, Adventure, and Practical Application: Virtual Reality in an Academic Health Sciences Library

**Published:** 2017-10-01

**Authors:** Kimberley R. Barker

**Affiliations:** Claude Moore Health Sciences Library, University of Virginia

The Claude Moore Health Sciences Library has a rich history of patron-centered technology projects, not surprising for an institution that includes innovation as part of its mission of supporting the education, research, patient care, and community service missions of the University of Virginia Health System. In this tradition, a small team closely monitored developments in virtual reality (VR) technology and its health care–related possibilities for several years and recently implemented a VR service for the library’s staff and patrons.

Planning for the project began in August 2016. Initial discussions focused on which VR system would meet the following requirements: best meets patrons’ needs, performs reliably, has the capability to run relevant simulations, and stays within budget restrictions. Other planning considerations included creating a budget for the entire project; setting up visits to other departments on campus to learn more about their VR services; canvassing the library to find a physical space to showcase the VR system; and discussing how best to prepare for issues of user safety, including considerations of tripping, falling, dizziness, and disorientation.

The VIVE system was selected, and the order was placed in September 2016, along with orders for a dedicated desktop computer, headphones, tripods for the base stations, and bumper guards for the dedicated VR space. While waiting for the order to arrive, the implementation team began preparing to teach workshops, which included conducting literature searches, scanning social media, seeking out collaborators, outlining the workshop agenda, creating a LibGuide for the new VR service, and developing an interest survey that would be given to workshop attendees.

The system arrived in October 2016 and was set up and eagerly tested by the VR implementation team. Set-up included installing the VIVE software, setting up the base stations, and outlining the VR “play area.” VR testing involved team members working in the simulations to become familiar with how to explain the technology to users and the best ways to move in the simulations, how to adjust the headset for maximum comfort and optimal performance, how to use the handset buttons in different simulations, and how to effectively communicate with patrons to ensure their safety. Initial demonstrations of the new VR system were offered exclusively to library faculty and staff on two different days. This enabled library staff to understand and use the new VR technology, and it provided a friendly audience for the team for practicing their instruction.

The technology involved in the project included an HTC VIVE kit (which includes headset, handheld peripherals, and base stations) and an Alienware Aurora desktop, as well as various simulations that ranged in cost from free to $29.00. Future technology purchases planned for the VR service include a wireless adapter for the headset (to reduce the tripping hazard) and haptic gloves to further immerse users in the VR experience.

The primary limitation for this project was that our efforts ended at the introductory level due to staffing and budget limitations. Users expressed an interest in working with VR coders to create their own simulations, but the library did not have sufficient staff to dedicate someone to work on VR development projects. While this was frustrating to the VR team, through networking and collaboration, the VR team was able to identify other departments that could address these coding needs and make referrals.

Between November 2016 and March 2017, the VR implementation team reached approximately 150 health system members through a combination of large-group demonstrations, one-on-one consultations, and workshops. All attendees of the workshops participated in a survey that focused on the potential uses of VR in health care. Interestingly, the majority of respondents believed strongly that VR was likely to impact medical education, patient education, and surgery simulation.

Overall, the library is pleased with the outcomes of the project. Its goal with VR, as with the introduction of any emerging technology, is to expose as many members of the health system to a technology that is increasingly important to their work, as well as to provide them with real-world examples of both how the technology is currently used and how it could be used in the future. The relatively low entry cost for this technology also made it appealing, as did the opportunity to network with other departments interested in VR. This project has led to the library’s participation in a pan-university initiative to further explore the technology. Survey data have yielded valuable insights into the interests of our patrons regarding VR, and the project has provided an opportunity to collaborate with the School of Medicine’s Simulation Center through employing a medical student as an intern for the library’s VR program. Lastly, the introduction of VR to the health system aided in maintaining the library’s reputation as a center for cutting-edge technology and teaching.

# Other: Is there Life on Mars? Gamification of Evidence-Based Practice Learning

**Published:** 2017-10-01

**Authors:** Grai Calvey, Heather Cooper, Mary Simons

**Affiliations:** Macquarie University Library, Macquarie University, Australia

The online *Evidence-Based Mars Mission* game was created as an innovative way to teach the basic concepts of evidence-based practice (EBP) to undergraduate students. The aim was to develop an enjoyable activity that uses a science fiction scenario to engage students in identifying different levels of evidence using EBP concepts. It was hoped that the online game format would appeal to most first-year university students, often referred to as the “gaming generation.” The game was developed to be a part of the year one EBP program that sits in the professional practice unit of a clinical science program.

Gamification has its foundations in experiential learning theory where play, motivation, challenges, rewards, and learning through doing enhance the learning experience [1]. Game theory is now widely believed to be a new pedagogy: “The centrality of enjoyment and engagement in gaming pedagogy makes it uniquely effective in situations where other forms of pedagogy may struggle” [2]. The authors wanted to ascertain if a game could increase learning of EBP study designs using an interactive approach, rather than traditional ways of didactic learning.

The project team, led by librarians, included clinical, educational, gaming, and information technology experts in the university. A nonhuman disease was included in the scenario so that students would have no prior knowledge of the illness. A pathogen that is transferred from bees to humans on the planet Mars formed an intriguing context.

The game required students to navigate each level of an evidence pyramid by successfully completing tasks that tested their knowledge of study designs before proceeding to the next level and eventually eradicating the disease. Tasks consisted of multiple choice questions, cloze tests, and matching activities (Figure 1). The game began with a challenge for students to join a mission to solve the problem of diminishing food supplies for the Mars population. The first few layers of a standard evidence-based medicine (EBM) pyramid became the game content, and the levels of the game were aligned with the levels of the pyramid (Figure 2). Learning was carefully scaffolded so that knowledge increased with each successive level. Every challenge was self-correcting, and multiple attempts were allowed. The length of the game was kept to a maximum of thirty minutes.

**Figure 1:** Cloze activity in the game

The reward for success is progression to the next level. At the successful conclusion of the game, an invitation is extended by the chief scientist (played by the associate dean of clinical medicine) to use EBP knowledge for the benefit of mankind. The final version was piloted with library staff and university students before being added to the online learning platform. Each student undertook the game after completing all the module’s related EBP learning activities. Upon completing the game, each student posted to an online forum a reflection on the value of the game to their EBP knowledge and to their future careers. Reflective feedback mostly indicated an appreciation of the game’s role in reinforcing EBP principles in a stimulating format.

**Figure 2:** Aligning game activities with the levels of the evidence-based medicine (EBM) pyramid

The library budget available for the project determined the software and expertise utilized. Library staff created video clips for the game scenarios using Adobe Captivate 8 software, which was compatible with the university Moodle platform. Internet Explorer 9 or later, Safari 5.1 or later, or Google Chrome 17 or later were required. The complex tasks of linking instructions, activities, and feedback were completed by an educational developer in the university.

Scenario development required creativity, knowledge of gamification principles, and appreciation of science fiction. The experience of game development helped the project team to increase their understanding of EBP and online gamification techniques. Student feedback indicated an awareness that EBP principles could be applied to a clinical scenario to solve a problem. While most student feedback was positive, some suggested ways to improve the game, such as increasing the level of difficulty and varying the kinds of tasks. Technical issues impeding the seamlessness of the game will be reviewed for the next version. Completion of the highest levels of evidence is also planned. This game has potential application to a range of health-related disciplines and levels of complexity, as well as supporting blended learning and flipped classroom activities.

The *Evidence-based Mars Mission* game, Level 1, is available online. *Evidence-based Mars Mission* by Macquarie University Library is licensed under a Creative Commons Attribution-NonCommercial-NoDerivatives 4.0 International License. Permissions beyond the scope of this license are available online.

## References

[1] Banfield J, Wilkerson B (2014). Increasing student intrinsic motivation and self-efficacy through gamification pedagogy. Contemp Issues Educ Res (CIER).

[2] Tulloch R (2014). Reconceptualising gamification: play and pedagogy.. Digit Cult Educ.

# Other: Using Data Dashboards to Teach Undergraduates to Visualize Health Inequities

**Published:** 2017-10-01

**Authors:** Porcia Vaughn, Michelle Catalano, Josh Been

**Affiliations:** Life Science Library, University of Texas at Austin;; Anderson Library, University of Houston;; Houston Independent School District

To promote data literacy skills among students in health sciences programs at the University of Houston, librarians and faculty in health education and nursing developed data dashboards. The data dashboards serve as experiential learning opportunities, which allow students to map out the future of health in their own communities. Students in these courses learn valuable data literacy skills that they can apply to their health professions as they enter the workforce.

Staff created learning objects, data dashboards, to give students a practical and realistic way to interact with a variety of complex datasets that could be used to target populations for specific health interventions. The librarians and faculty worked together to create course activities, which required students to identify, interpret, and communicate data as a means to provide health education to an at-risk population. The library was strategically positioned to support building data dashboards by employing a data librarian with a specialty in geographic information systems (GIS) and data visualizations.

To develop the interactive dashboards, we created a five-step plan: (1) create a data management plan, (2) collect data sources, (3) analyze data, (4) wrangle data, and (5) build visualizations. The data management plan was created for each course project, as we would be working with a variety of data sources. Depending on the needs of the course, we created dashboards containing several data points that would need to be seamlessly managed during the data dashboard creation. It was important to us to be able to track raw data sources and to preserve unique data dashboards as a means to be transparent, to be reused, and to replicate pieces of the dashboards to create other learning objects or to advance ongoing research initiatives in health sciences and community health.

Data collection included a variety of data sources, both publicly available and proprietary. Data sources depended heavily on the instructor’s main focus, in other words, data related to lead exposure risk or set-up locations for clinics. Data points and variables were collected from the US Census, US Health Resources and Services Administration (HRSA), US Department of Agriculture (USDA), Texas Education Agency (TEA), the National Historical Geographic Information System, crime reports, Reference USA, Healthy People 2020, or in class-generated survey data.

Data analysis and normalization took place using QGIS and Trifacta Wrangler to identify patterns and stories in the data that would be highlighted in the interactive visualizations. Once the data were cleaned and normalized, we used Tableau Public to build interactive visualizations. We embedded some of these visualizations into course LibGuides, while others remained in the Tableau Public account dashboard and were shared using a persistent link.

In the LibGuide for the “Community Health Nursing” course, the students were able to conduct a suitability analysis for lead exposure by giving a weighted value to each variable, thereby creating a risk index and identifying vulnerable populations.

In another data dashboard project for the “Social Health & Wellness” course, the students viewed two health variables in Texas counties to determine what type of relationships existed between the health indicators. Students were then asked to find supporting literature that explained reasons for these possible relationships. In both cases, data dashboards gave the students the ability to select and weight variables, empowering them to make better informed, evidence-based decisions in their own communities, which typically does not happen in the course curriculum.

Interest in data dashboards continues to grow among faculty in other disciplines. Recently, the university embarked on five-year quality enhancement plan (QEP) to improve student learning through incorporating real-world and project-based experiences into the undergraduate curriculum. The library’s success with developing and utilizing data dashboards uniquely positions it to play an important role in achieving the university’s goal of providing experiential learning opportunities.

# Other: A Repository Solution for the Future

**Published:** 2017-10-01

**Authors:** Violeta Ilik, Kristi L. Holmes

**Affiliations:** Feinberg School of Medicine, Northwestern University

The Northwestern University Feinberg School of Medicine’s Galter Health Sciences Library & Learning Center (GHSLLC) launched a next-generation repository to support the preservation and dissemination of research and scholarship. The DigitalHub institutional repository provides Northwestern Medicine with stable, long-term storage and ongoing maintenance for a wide range of scholarly products. The flexible repository can meet evolving needs and has emerged as a strong tool to support dissemination and discovery. The Feinberg School of Medicine is research focused, generating 68% of all research dollars at Northwestern University and growing steadily in National Institutes of Health (NIH) research funding. This research focus continues in Feinberg’s strong education and patient care mission, with medical students involved in individualized independent projects and over 50,000 patients and volunteers actively engaged in research studies at Feinberg each year.

DigitalHub provides needed infrastructure to support the research-intensive mission of the school. Like other repositories, DigitalHub houses published research articles but is uniquely focused on nontraditional scholarly outputs created during the research process by faculty, staff, and students to provide open access to diverse knowledge. By providing access and stewardship for nontraditional scholarly outputs, GHSLLC aims to expand and diversify the scholarly publishing and communication ecosystem. GHSLLC aligns this goal with the Confederation of Open Access Repositories (COAR) vision that repositories need to provide access to published articles, as well as a broad range of artifacts beyond traditional publications. Examples of items that the repository can accommodate include research papers (published or unpublished), case reports, technical reports, supplemental images, datasets, open access books, conference papers, white papers, presentations and lectures, posters, and video and audio materials. Materials are made available with a Creative Commons license, and the Massachusetts Institute of Technology (MIT) license is available for software packages and supporting materials.

The DigitalHub repository was launched in October 2015. DigitalHub is built on the Fedora 4.5 and Sufia 6.6 digital asset repository platform. We use an International Image Interoperability Framework (IIIF) server and OpenSeadragon image viewer to make our image resources available to web applications hosted on other domains. Submitting an item to DigitalHub is easy, taking seven steps with five required fields. Three of the required fields are controlled: resource types, using the National Library of Medicine publication type list; creator name, verified against the Northwestern University directory; and the rights statement. We use established standards (date-ISO 8601 and language-ISO 639-2) and established controlled vocabularies (Library of Congress Subject Headings [LCSH], Medical Subject Headings [MeSH], and GeoNames). To ensure reliable data management, data sharing, and citation tracking, we assign persistent unique identifiers (digital object identifiers [DOIs] and archival resource keys [ARKs]).

The GHSLLC Digital Initiatives Working group continues to develop the repository based on user feedback, such as supporting large file deposit, video and audio file upload, and additional metadata fields that capture funding information and acknowledgments. Our future plans include connecting the repository to a metadata aggregator that alerts users when open access journal articles by Feinberg faculty are available, followed by their automatic deposit into DigitalHub.

# Other: Medline Transpose: Conversion Tool for Pubmed and Ovid Medline Interfaces

**Published:** 2017-10-01

**Authors:** Amanda Wanner, Niki Baumann

**Affiliations:** University of Plymouth, United Kingdom;; College of Physicians and Surgeons of British Columbia (CPSBC), Canada

MEDLINE Transpose was developed to mitigate a common problem: converting search syntax between the PubMed and Ovid MEDLINE interfaces (Figure 3). When formulating a complex search strategy involving free text, controlled vocabulary terms, and field codes, searching both PubMed and Ovid MEDLINE can allow researchers to capitalize on the strengths of each, such as the adjacency (ADJ) function in Ovid or the unique PubMed Central (PMC) content in PubMed. An increasing amount of research calls for researchers to search both interfaces [1]; however, converting a search strategy for use in the opposite interface can be a complex process [2].

Field codes and interpretation of user input differ between Ovid and PubMed. For example, both the “text word” and “title/abstract” field codes search different combinations of fields in PubMed and Ovid MEDLINE, despite using the same terminology. Therefore, translating between interfaces can be time consuming and requires thorough consultation of the support documentation, in particular where no exact translation is possible. Likewise, in a search strategy with many terms, it can be tedious to make the same change repeatedly, but conversion is often beyond the capability of a simple find-and-replace strategy. PubMed, for example, requires each term to be followed by its field code, while Ovid allows a shorthand syntax with field codes succeeding a group of search terms.

While automating search translation between Ovid and PubMed has previously been attempted, these attempts have relied on a simple find-and-replace strategy. MEDLINE Transpose uniquely converts strategies, while allowing variety in the ways that Medical Subject Headings (MeSH) or keywords can be searched in each. The conversion tool also strives for the most accurate conversion possible and provides warnings and suggestions where an exact conversion is not possible. This allows the user to make informed decisions about the best possible conversion strategy.

**Figure 3:** MEDLINE Transpose interface

MEDLINE Transpose was hand-coded by the authors using JavaScript, and the interface was built using W3.CSS, a freely available and modular system that can be infinitely customized. The program is hosted on the GitHub.com development platform, a repository and hosting service for open source code, and the code is publicly available to view, modify, and reuse under the MIT license .

Some applications of MEDLINE Transpose include:

- more accurate and efficient way of searching both PubMed and Ovid MEDLINE for a systematic review
- ability to capitalize on the strengths of both databases during day-to-day use, such as quickly converting a draft search string into Ovid syntax to use the adjacency operator or into PubMed syntax to use a pharmacological action concept
- ability to use another person’s or institution’s premade search strategy in the alternate interface; for instance, a search hedge

There are limitations to the translation abilities of MEDLINE Transpose. Most prominently, not all searches have an exact translation, and in these cases, the program can suggest alternatives but cannot create database functionalities that do not currently exist. The program also relies on user input that is formatted correctly. Because MEDLINE Transpose looks for patterns, rather than reading and understanding the input content, it cannot identify spelling mistakes, retired MeSH, or incorrectly formed syntax (although some error corrections have been incorporated into the program).

Conversions for the most commonly used search features of MEDLINE have been implemented. These have undergone extensive usability testing, both by the authors and a group of medical librarians identified through snowball sampling. Results from usability testing have indicated that the program has worked as intended. Users have also suggested several features for future implementation. Based in part on this feedback, planned improvements to MEDLINE Transpose include: a multiline entry field, recognition of tagless “Filter” style limits in PubMed and codeless limits in Ovid syntax, and the option to choose between the most accurate and a more efficiency-based conversion. The authors plan to keep abreast of changes to the search capabilities of both databases and implement changes as necessary.

MEDLINE Transpose is freely available online.

## References

[1] Duffy S, de Kock S, Misso K, Noake C, Ross J, Stirk L (2016). Supplementary searches of PubMed to improve currency of MEDLINE and MEDLINE In-Process searches via Ovid.. J Med Libr Assoc.

[2] Damarell RA, Tieman JJ, Sladek RM (2013). OvidSP Medline-to-PubMed search filter translation: a methodology for extending search filter range to include PubMed’s unique content.. BMC Med Res Method.

# Other: Designing and Implementing a Microsoft Access Database for Patron Interaction Tracking: A Budget-Friendly Reference Statistics Solution

**Published:** 2017-10-01

**Authors:** Lydia Howes

**Affiliations:** School of Medicine Library, Southern Illinois University

Tracking, managing, and analyzing reference statistics is essential for libraries, but the process can be burdensome and commercial solutions are expensive. At the Southern Illinois University (SIU) Medical Library, staff had been tracking desk statistics using tick marks on paper sheets, which were periodically transcribed to Microsoft Excel. This process, which had been in place for many years, was not only time consuming, but also failed to capture the nature of multifaceted interactions.

We wanted to automate and upgrade our system, but budget constraints eliminated commercial products, and free web-based solutions, such as Google Forms, would not improve upon Excel’s abilities. Ultimately, we decided to create our own patron interaction (PI) database using Microsoft Access because a well-designed database can easily manage and organize complex data and more accurately capture multifaceted interactions than our previous system could. Microsoft Access is a database management system included in the Microsoft Office suite of programs that provides greater flexibility than Excel. Because the Microsoft suite was already licensed by our institution, the use of Microsoft Access incurred no additional costs.

Identifying the information that the library wanted to track was the first step in designing the database and user input form. We first included data reported to outside organizations, such as the Association of College and Research Libraries (ACRL) and Association of Academic Health Sciences Libraries (AAHSL) and then held informal discussions with staff to identify their needs and interests. Date and time are automatically recorded when new records are added, and staff is only required to supply the method of contact and patron affiliation with SIU. Although further descriptors—including directions, instruction, mediated searching, technology support, equipment lending, and a “notes” field—are optional, the vast majority of entries include at least one non-mandatory descriptor.

The database is separated into two sections, saved as separate files. This front-end and back-end arrangement allows multiple people to input data at once, while protecting tables from unintentional edits or deletions and retaining some customization. The “back-end” is stored on the library’s shared network drive and includes all the underlying tables and stored data. The “front-end” contains all forms and queries, including the PI user input form (Figure 4) and a “Quick Stats” section that allows staff to quickly pull data from tables (Figure 5).

The “Stat Digest,” one of the queries included in “Quick Stats,” generates tallies of various types of patron contacts (Figure 6). It is based on reports that the library provides to ACRL and AAHSL. Other queries can pull records for all interactions that match the designated parameters. These datasets can then be saved for further analysis in Access or exported for use in other programs. Each staff member has a copy of the front-end saved to their workstations and uses the forms to easily add entries to or pull data from the PI database as needed.

Beta testing lasted from July 2016 to October 2016 and involved recording interactions using both the old tick mark method and the PI database. During this period, we added several descriptors and fixed small bugs. Testing ended once we were satisfied that the form worked correctly. We added the Quick Stats section a few months after initial beta-testing had ended.

**Figure 4:** Patron input form

**Figure 5:** Quick Queries interface

**Figure 6:** Reference statistics report

Even though database development, testing, and maintenance require a significant time investment, the SIU Medical Library is satisfied with the project’s outcome. The PI database makes inputting, accessing, and interpreting data easier and has already been useful in decision making. We already knew that patron interactions with staff were extremely rare on weekends, but the new, more robust statistics confirmed our suspicions that nearly all those interactions involved either providing directions or computer support to persons who were unaffiliated with the medical school. This new information, along with the fact that a recent renovation allows the library to provide 24/7 secure study access for students, supported the decision to reduce staff hours by closing the library on weekends.
